# Design and Implementation of an Underwater Sound Recording Device

**DOI:** 10.3390/s110908519

**Published:** 2011-09-01

**Authors:** Jayson J. Martinez, Josh R. Myers, Thomas J. Carlson, Z. Daniel Deng, John S. Rohrer, Kurt A. Caviggia, Christa M. Woodley, Mark A. Weiland

**Affiliations:** Pacific Northwest National Laboratory, P.O. Box 999, Richland, WA 99352, USA; E-Mails: Jayson.Martinez@pnnl.gov (J.J.M.); joshua.myers@pnnl.gov (J.R.M.); thomas.carlson@pnnl.gov (T.J.C.); john.rohrer@pnnl.gov (J.S.R.); kurt.caviggia@pnnl.gov (K.A.C.); christa.woodley@pnnl.gov (C.M.W.); mark.weiland@pnnl.gov (M.A.W.)

**Keywords:** underwater sound recording, underwater acoustics, blasting

## Abstract

To monitor the underwater sound and pressure waves generated by anthropogenic activities such as underwater blasting and pile driving, an autonomous system was designed to record underwater acoustic signals. The underwater sound recording device (USR) allows for connections of two hydrophones or other dynamic pressure sensors, filters high frequency noise out of the collected signals, has a gain that can be independently set for each sensor, and allows for 2 h of data collection. Two versions of the USR were created: a submersible model deployable to a maximum depth of 300 m, and a watertight but not fully submersible model. Tests were performed on the USR in the laboratory using a data acquisition system to send single-frequency sinusoidal voltages directly to each component. These tests verified that the device operates as designed and performs as well as larger commercially available data acquisition systems, which are not suited for field use. On average, the designed gain values differed from the actual measured gain values by about 0.35 dB. A prototype of the device was used in a case study to measure blast pressures while investigating the effect of underwater rock blasting on juvenile Chinook salmon and rainbow trout. In the case study, maximum positive pressure from the blast was found to be significantly correlated with frequency of injury for individual fish. The case study also demonstrated that the device withstood operation in harsh environments, making it a valuable tool for collecting field measurements.

## 1. Introduction

Many underwater processes, such as underwater blasting or pile driving, have the potential to create sound and pressure waves that harm fish or other aquatic life [1–7]. Fish injuries documented from such activities range from non-lethal to lethal depending on sound and pressure wave characteristics. Non-lethal injuries experienced by fish could result in altered physiological states, including changes in scale loss, hormone levels, sensory detection, stress and/or immune responses, tissue damage, embolisms, and ruptured swim bladders, which may manifest as changes in behaviors and performance [5,8–11]. Altered perception of threats and subsequent behavioral changes can increase the risk of exposure to predation by piscivorous fish, marine mammals, and birds [12,13]. In addition, non-lethal injuries could manifest as decreased performance, particularly swimming performance in which fish struggle to maintain buoyancy and equilibrium and to avoid obstacles or unfavorable water flow [12,14,15]. Lethal injuries are typically the result of major injuries (e.g., gills and/or heart embolisms, internal organ damage or lacerations), or the culmination of mild and moderate injuries that result in high blood loss or major tissue damage [1,16].

Given the diversity of underwater activities, environments (e.g., open or closed water, substrate type, ambient noise) in which these activities are conducted, and species-specific biology, the scientific community does not completely agree on which or how acoustical sound and pressure wave characteristics drive the effects seen in fish. While the chemical and physical parameters of underwater sound and pressure waves are fairly well understood, the resultant sound and pressure waves are complex [17]. To unravel the complexity of these waves and their effects on fish, information is needed on explosives (e.g., charge and blast timing, charge and blast weights, rock type, and explosion design features such as number, depth, and spacing of holes) or exposure characteristics (e.g., frequency of strikes, intensity, duration, and other parameters), and their energy releases (e.g., amplitude, frequency, duration, pressure, impulse, energy flux density) [17–19].

Monitoring potentially harmful underwater processes requires a portable, autonomous, watertight, and user-friendly device (operable by non-engineers or technicians) that can be connected to multiple hydrophones or other underwater sound/pressure sensors. The device also must tolerate handling in extreme environments (e.g., exposure to weather, small boats loaded with gear). Hydrophones can be used to measure dynamic pressure changes but typically output relatively low voltages, so the incoming signals often need to be amplified. Thus, the device should have an amplifier with a variable gain control to allow for measuring underwater acoustic signals with a wide range of sound pressure levels. The same device could be used also for other activities such as recording sounds generated by vessels or the vocalizations of some marine mammals.

There are several state of the art autonomous acoustic recording devices built by various research institutes [20–23] and commercially available systems or other aquatic sensor networks [24]. However, they were either designed for ocean environment and applications (ocean noise or marine mammal detections), or did not meet our requirements for sampling frequency, portability and cost, or did not offer the flexibility of switching hydrophones and underwater sound sensors for different environments or underwater processes. The purpose of this study was to design, build, and test a deployable underwater sound recording device (USR) to study underwater processes (such as underwater blasting or pile driving) and their potential impact on fish or other aquatic life. The USR is a portable, watertight, and easy-to-use device designed to record underwater acoustic signals from two hydrophones or other underwater sound sensors. Two models of the USR were designed—a submersible model and a watertight yet non-submersible model. Bench top laboratory testing verified that the device operates as designed. Simulated field testing performed in a large water tank using a hydrophone and a high-power underwater transducer demonstrated that the USR performs as well as larger commercially available data acquisition systems, which are not suited for field use. The USR was used in a case study where juvenile Chinook salmon and rainbow trout were exposed to sound and pressure waves resulting from the underwater rock blasting performed to deepen the navigation channel in the Columbia River between Portland, Oregon, USA, and the Pacific Ocean.

## 2. Design and Implementation

**Figure 1.**
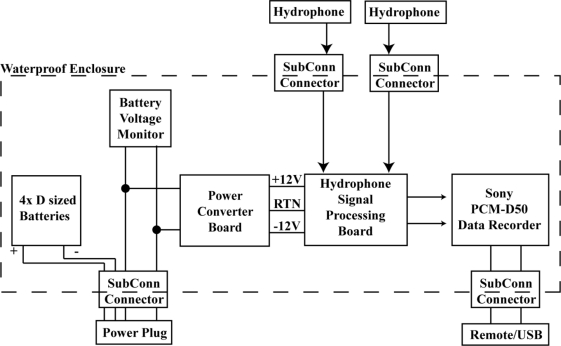
Underwater Sound Recorder schematic.

The USR consists of four D-cell batteries, battery voltage monitor, power converter board, sensor signal processing board, digital data recorder, watertight housing, two waterproof connectors for sensors, one waterproof connecter for a wired remote control, and a fourth waterproof connector for enabling power (Figure 1). Two different types of housing are available—one for data acquisition where the data can be accessed at the surface (non-submersible) and another designed to be completely submersed in water up to a depth of 300 m.

### 2.1. Power Converter Board

**Figure 2.**
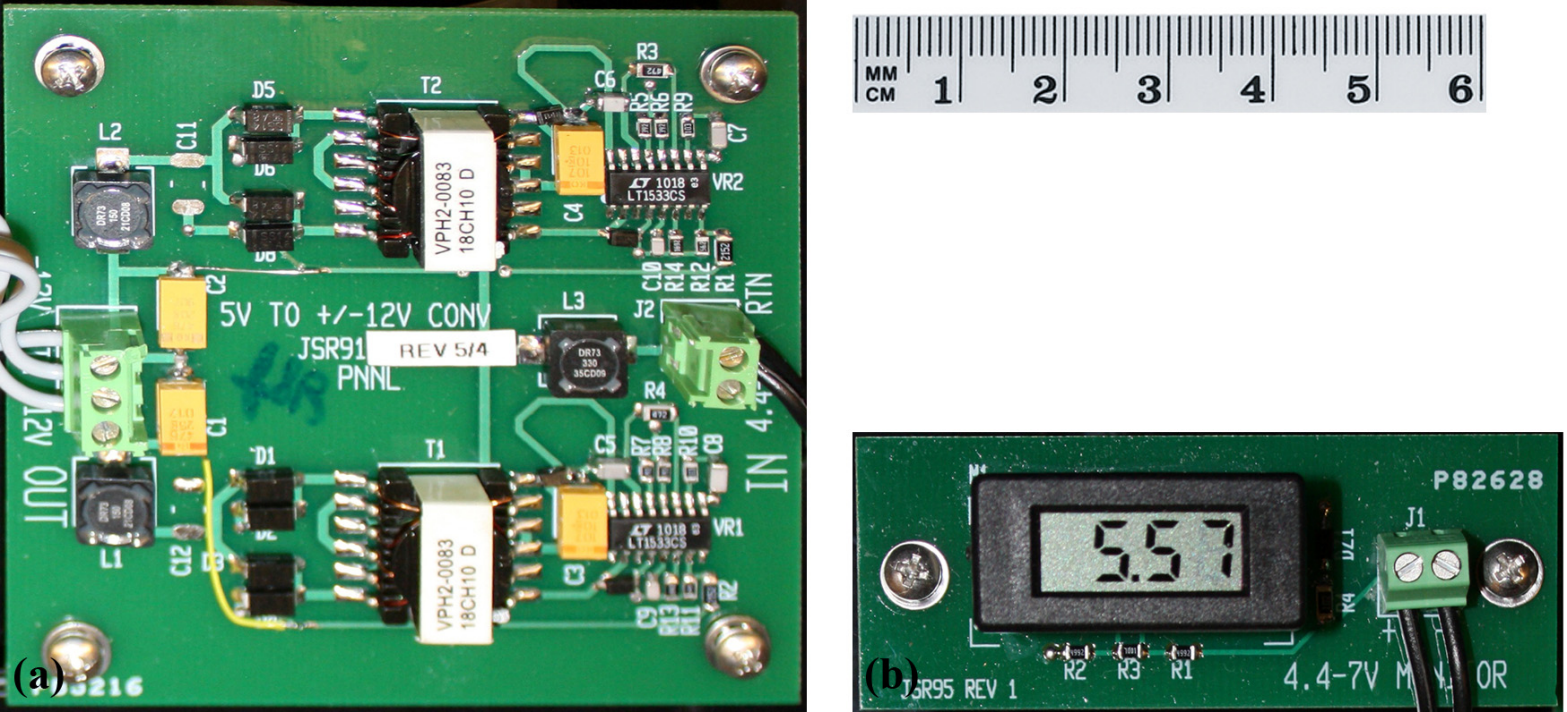
Power conversion unit. (**a**) Power converter board; (**b**) battery voltage monitor.

The power converter board [Figure 2(a)] converts the voltage from the four D-cell batteries to ±12 V. Voltage conversion is accomplished by two switching regulators (Model LT1533 1A, Linear Technology, Milpitas, CA, USA). These regulators were chosen for their fast slew rate. The circuit design achieves a system efficiency of approximately 35%, which results in a battery life of approximately 30 h. The input voltage range of the circuit is 4.4 to 7 V, enabling the use of either alkaline or nickel–metal hydride (NiMH) batteries.

### 2.2. Battery Voltage Monitor

The battery voltage monitor circuit board [Figure 2(b)] displays the current battery voltage on a small 3.5-digit liquid crystal display (Model DPM 1AS, Martel Electronics Corporation, Londonderry, NH, USA). The display enables the user to easily assess battery condition by viewing current battery voltage to determine whether the batteries need to be changed.

### 2.3. Hydrophone Signal Processing Board

**Figure 3.**
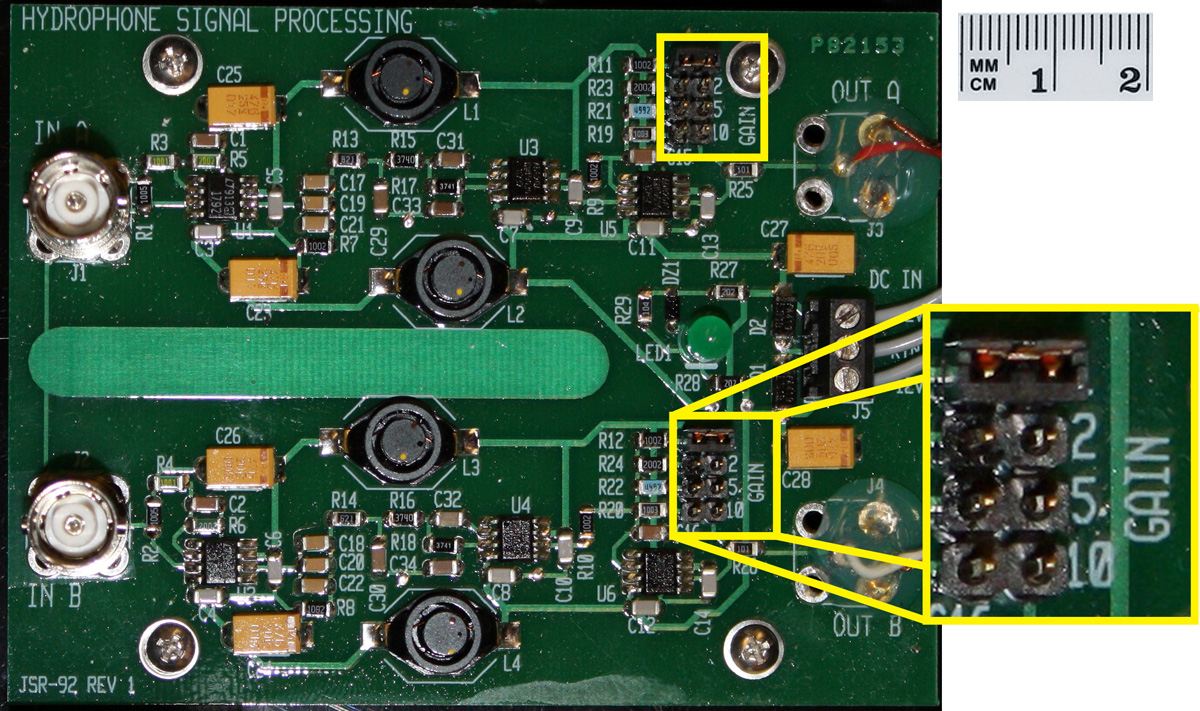
Hydrophone signal processing board. The gain selector jumper pins for each channel are highlighted in yellow.

The hydrophone signal processing board is designed to process the signals from two hydrophones or other sensors in parallel. Each signal is initially amplified by a 26-dB preamplifier. The signals then pass through a three-pole low-pass Bessel filter [25] with a cut-off frequency (−3 dB) of 15 kHz. A Bessel filter was chosen for its ability to preserve the shape of the filtered waveform due to its flat group delay. After passing through the filter, the signal goes through a gain stage with user-selectable gain values of 1×, 2×, 5×, or 10× (0 dB, 6 dB, 14 dB, or 20 dB). The amount of additional gain for each channel is selected by placing a jumper across the appropriate pins (Figure 3).

### 2.4. Data Recorder

The filtered waveforms are saved to a digital data recorder (Model PCM-D50, Sony Corporation, Tokyo, Japan). The data recorder features a maximum resolution of 24 bits and a maximum sampling rate of 96 kHz. The data recorder has 4 gigabytes (GB) of built-in memory, which allows a total recording time of approximately 1 h and 55 min at the maximum resolution and sampling rate. The total amount of memory can be expanded by up to an additional 4 GB by using a Memory Stick Pro-HG Duo memory card (Sony Corporation), although only one type of memory may be used at a time. To start and stop recording with the data recorder from outside the USR enclosure, a wired remote control module (Model RM-PCM1, Sony Corporation) is included. The data are saved to the memory as waveform audio files (xxx.wav, also referred to as “wave” files), which can be downloaded from the data recorder through a universal serial bus (USB) connection with a personal computer (PC). To ensure that the gain of the USR is well defined, the knob that controls the recording gain of the data recorder is set to its maximum value and a piece of tape is placed over the knob to prevent the user from unintentionally moving it. At the maximum recording gain, the data recorder provides about 4 dB of gain.

### 2.5. Non-Submersible Housing

**Figure 4.**
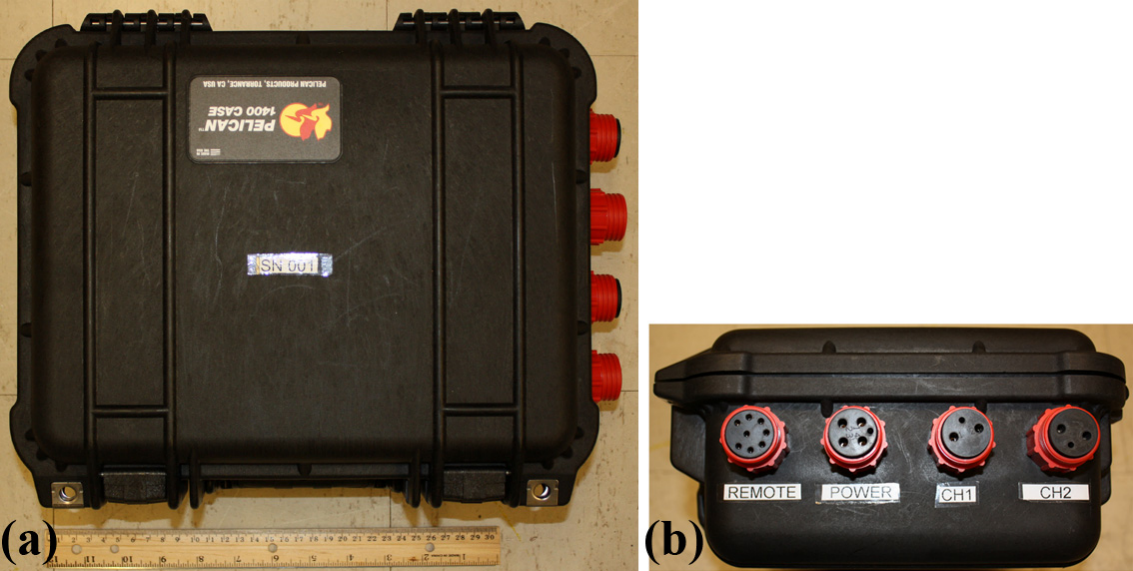
Non-submersible version of the Underwater Sound Recorder. (**a**) Pelican 1400 case; (**b**) external waterproof connections.

A Pelican 1400 Case (Pelican Products Inc., Torrance, CA, USA) is used for the non-submersible version of the USR (Figure 4). The outside dimensions of this case are 34.0 × 29.5 × 15.2 cm (excluding connectors). On the side of the case are four underwater mateable connectors (SubConn Inc., Burwell, NE, USA). Two of the connectors are used for connecting each of the hydrophones. If only one hydrophone is to be used, a submersible plug is included to keep the connector terminals dry. Another connector is used for powering up the device by inserting a submersible plug. The fourth connector is for connecting the remote control module, whose cable also includes the USB connector. Similar to the hydrophone connectors, when the remote and USB connection are not needed, a provided submersible plug may be used in its place.

### 2.6. Submersible Housing

**Figure 5.**
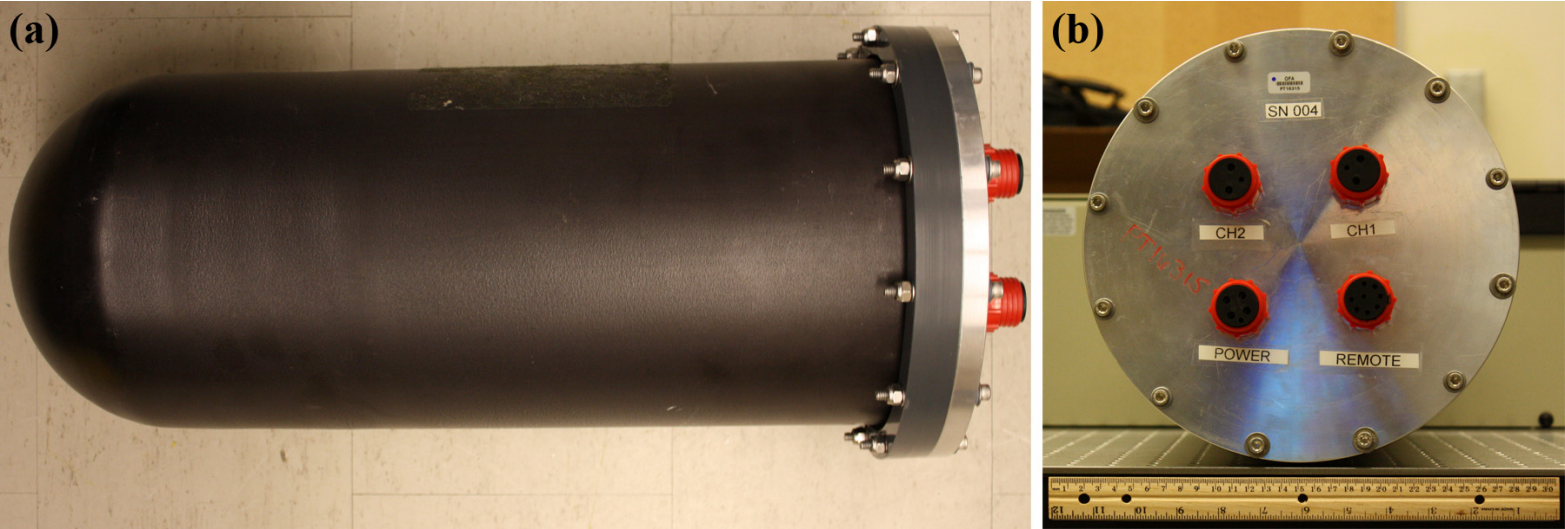
Submersible version of the Underwater Sound Recorder. **(a)** PREVCO submersible housing; **(b)** external waterproof connections.

For the submersible version of the USR, a submersible housing (Model A8.403SS, PREVCO Subsea LLC, Fountain Hills, AZ, USA) is used (Figure 5). The housing is constructed from aluminum and is rated for a maximum depth of 300 m. The maximum outside diameter is 26.7 cm, and the length (excluding connectors) is 59.0 cm. The mass of the housing is 10.9 kg in air and −10.3 kg in seawater. SubConn underwater mateable connectors, identical to the ones used on the non-submersible version of the USR, are also used for connecting the hydrophones, remote control module and USB, and for turning on recorder power.

## 3. Testing Methods

To test the hydrophone signal processing board, the PCM-D50 data recorder, and the complete USR system, single-frequency sinusoidal voltages were supplied by a data acquisition system, described in subsequent sections, directly to the input of the component being tested. Signals at fifty logarithmically spaced frequencies between 100 Hz and 50 kHz were sent to the recording system component being tested. Each signal was composed of 1,000 cycles per frequency, with 100 sample points per period, except when limited by the maximum sampling rate, which occurred at frequencies higher than 40 kHz. After signals at each frequency were sent, the results were analyzed by calculating the root mean square voltages of the signals’ input and output from the USR element being tested using Equation (1).

(1) $$V_{\mathrm{RMS}}=\sqrt{\frac{\sum_{i=1}^{n}x_{i}^{2}}{n}}$$

The gain in dB re 1 V through the USR element at the test frequency was calculated using Equation (2).

(2) $$\mathrm{Gain}=20*{\text{log}}_{10}\left(\frac{V_{\mathrm{RMS\_out}}}{V_{\mathrm{RMS\_in}}}\right)$$

Four different USRs, two of each design, were tested.

### 3.1. Hydrophone Signal Processing Board

The USR hydrophone signal processing boards were tested using a data acquisition card (Model PXIe-6124, National Instruments [NI], Austin, TX, USA) housed in a PXIe-1073 chassis (NI) and connected to a computer running the Microsoft Windows 7 operating system. The NI data acquisition card has a 16-bit analog-to-digital converter (analog input) and a 16-bit digital-to-analog converter (analog output), each with a maximum sampling rate of 4 MHz. The data acquisition card was controlled by a MATLAB program (The MathWorks, Inc., Natick, MA, USA) written specifically for these tests. All data analysis also was conducted using MATLAB programs. One of the PXIe-6124 analog outputs was used to send sinusoidal signals, with a peak-to-peak voltage of 90 mV, to one channel of the hydrophone signal processing board. The input of the channel not being tested was terminated with a 50-ohm load. The output of the hydrophone signal processing board was sent to one of the PXIe-6124 analog inputs.

The MATLAB program created to test the hydrophone signal processing board enabled completely automated testing of the board. After the program was run, the gain at each frequency was determined; in addition, the gain as a function of the input frequency was graphed. Each channel was tested at each user-selectable gain value. To accommodate each of the gain values, the analog input range of the data acquisition card was increased for the increasing amounts of gain. For the 1× gain selection, a ±1 V range was used; 2× was ±2 V, 5× was ±5 V, and 10× was ±10 V.

### 3.2. Data Recorder

The PCM-D50 data recorder also was tested using the NI PXIe-6124 data acquisition card. One of the PXIe-6124 analog outputs was used to send sinusoidal signals, with a peak-to-peak voltage of 1.2 V, to one channel of the PCM-D50 data recorder. The other channel of the data recorder was terminated with a 50-ohm load. Each input signal at each test frequency was recorded to a separate waveform audio file on the data recorder. This was accomplished by the MATLAB program prompting the tester when to begin recording and when to stop recording each test frequency signal to the data recorder. After all of the files were collected, they were transferred for post processing from the data recorder to a computer running MATLAB, through a USB cable connecting the data recorder and PC.

### 3.3. Complete Underwater Sound Recorder System

The test of the complete USR system followed a process similar to the test for the PCM-D50 data recorder alone. The main difference was the voltage of the sinusoidal signals input to the USR. The input voltage at higher USR gain settings had to be kept low to prevent clipping the signals when they were recorded by the PCM-D50 data recorder. Low voltages with high resolution were generated by attenuating a higher voltage signal from an NI data acquisition card using a Kay Model 837 attenuator (Kay Elemetrics Corp., Lincoln Park, NJ, USA). The NI PXIe-6124 was replaced by an NI PCI-6110 data acquisition card for this test to match the impedances of the attenuator and data acquisition card. The PCI-6110 has a 12-bit analog-to-digital converter with a maximum sampling rate of 5 MHz and a 16-bit digital-to-analog converter with a maximum sampling rate of 4 MHz. The hydrophone signal processing board test was performed on a board using the PCI-6110 data acquisition card and the results compared to those obtained using the NI PXIe-6124 card. Nearly identical results were obtained using both NI cards. This test ensured that the change in NI card would not affect test results. The peak-to-peak voltage of sinusoidal signals output from the PCI-6110 was 50 mV. The attenuation set on the Kay 837 attenuator was the same as the gain value set on the hydrophone signal processing board for the test being performed (0 dB, 6 dB, 14 dB, or 20 dB).

### 3.4. Tank Testing

**Figure 6.**
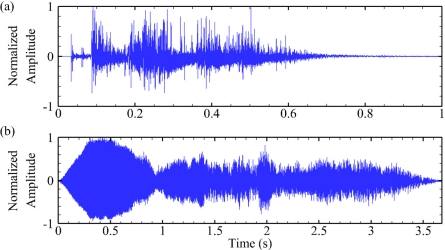
Waveforms used for tank testing. (**a**) Underwater explosion; (**b**) whale call.

Benchtop testing of the USR was followed by in-water tank testing. These tests were conducted to simulate real-world underwater acoustic signals. Two underwater acoustic signals were used, where the normalized waveforms are shown below (Figure 6). The first was an underwater explosion [16], and the second was a whale call [26]. The testing was performed in an elongated oval tank measuring 7.32 m long × 3.05 m wide × 1.83 m deep. The signals were saved as wave files and were sent from the audio output of a PC to an amplifier (Model IPA 300T, Architectural Acoustics, Corby, Northamptonshire, UK) that was connected to a high-power broadband piezoelectric underwater transducer (Model LL9162T, Lubell Labs Inc., Columbus, OH, USA). The underwater transducer was suspended at one-half the water depth along the centerline of the tank about 1.5 m from one end. A hydrophone (Type 8104, Brüel & Kjær, Copenhagen, Denmark) was placed in-line with the underwater transducer 1 m away.

The USR recorded each waveform at least five times for each channel and gain combination for each of the four USRs tested. For each gain value set on the USR, the gain of the IPA 300T was adjusted to avoid any clipping by the PCM-D50 data recorder. Immediately after the USR recorded each of the waveforms, the Type 8104 hydrophone was connected to a VP2000 amplifier (Reson Inc., Slangerup, Denmark) that was, in turn, connected to one of the analog inputs on the PXIe-6124 data acquisition card. The hydrophone and data acquisition system were calibrated in a tank lined with anechoic material [27]. With the same gain set on the IPA 300T amplifier, each waveform was recorded to the computer at least five times using the PXIe-6124 data acquisition card. To prevent any clipping of the waveform, the gain of the VP2000 amplifier and the input range on the PXIe-6124 were adjusted for each signal recorded.

After the signals were recorded using both the USRs and the PXIe-6124 data acquisition card for each USR channel and gain combination, the recorded waveforms were scaled using the corresponding measured gain of the USR and the measured gain of the VP2000 amplifier. The sensitivity of the hydrophone was then used to convert the units from voltage to pressure. With the waveforms scaled and converted to pressure units, a fast Fourier transform [28] was performed on each waveform and the resulting spectra were normalized by the waveform length to determine the frequency content of the recorded signals in physical units of pressure. The results were averaged for each set of measurements. Finally, the maximum pressure from the spectrum for the results obtained from the USR was compared with the results from the PXIe-6124 data acquisition card.

### 3.5. Case Study: Blasting Monitoring

Underwater rock blasting was one of the final steps to complete the 20-year Columbia River Channel Improvement Program that deepened the navigation channel between Portland, Oregon, and the Pacific Ocean to allow fully loaded Panamax ships (30 m wide, 183–213 m long, and 14–15 m draft) to transit safely. Regulatory agencies such as the National Oceanic and Atmospheric Administration do not permit underwater rock blasting (or blasts near aquatic environments) without mitigation of adverse sound and pressure effects. To monitor fish “take” from the blasting activities, direct mortality is the only response state readily detectable; however, the assessment can be conducted only if fish are recovered and examined immediately following exposure. Given the turbidity and outgoing flow rate of the Columbia River, heavy vessel traffic, and large monitoring area, it would be difficult to detect mortally injured or terminated fish from blast exposures [16]. In addition, not all injuries resulting from sound and pressure waves are fatal but require time for expression of nonlethal effects such as reduced fitness or increased susceptibility to predation.

Exposures of the caged fish (juvenile Chinook salmon and rainbow trout) were conducted to assess the effects on fish exposed to different sound and pressure waves created by the underwater rock blasting. The fish in exposure cages [16] were transported by boat to the predetermined coordinates provided by a U.S. Army Corps of Engineers representative. Cages were deployed into the water suspended from an anchored barge that held the pressure transducer systems and an air pump connected to a deep-cycle battery. The pressure transducer system consisted of ICP pressure sensors (Model 138A01, PCB Piezotronics, Depew, NY, USA), cabling, a power amplifier, a prototype version of the USR, and external file storage. All transducer data was adjusted for each individual transducer’s calibration. Once deployed, the system functionality was verified by banging the vessel and monitoring the signal on the digital recorder. Depending on the time, the recorder was either left in the Record position or paused for later start. All paused signal recording units were started a minimum of 15 min prior to the blast.

Exposure cages and pressure transducer systems were retrieved within 15 min of the blast and returned to the mobile lab at the marina for necropsy and system download. The necropsies involved careful observation of external and internal injury, based on 77 potential injuries. Sham control fish were handled, deployed, and retrieved similarly to blast-exposed fish except that their cages were redeployed to shore before detonation. After the data from the USR were downloaded, the signals were scaled to physical units using the measured system frequency response and the pressure transducer’s calibration. A fast Fourier transform was performed on each waveform [28], and the resulting spectra were normalized by the waveform length to determine the frequency content of the recorded signals in physical units of pressure.

## 4. Testing Results and Discussion

### 4.1. Hydrophone Signal Processing Board

**Figure 7.**
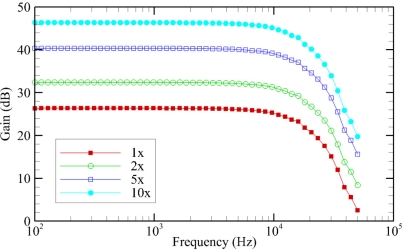
Average hydrophone signal processing board frequency response for each gain setting.

The average gain of the hydrophone signal processing board at each of the frequencies tested, for each of the gain settings on the hydrophone signal processing board, are plotted below (Figure 7). The observed gain of the hydrophone signal processing boards was on average approximately 0.35 dB higher than the designed values. Between the four different hydrophone signal processing boards tested, the gain values measured were very similar, with an average standard deviation of only 0.02 dB. The cutoff frequency for the low-pass filter, defined as the point at which the gain has dropped by 3 dB, had an average value of 15.1 kHz.

### 4.2. Data Recorder

**Figure 8.**
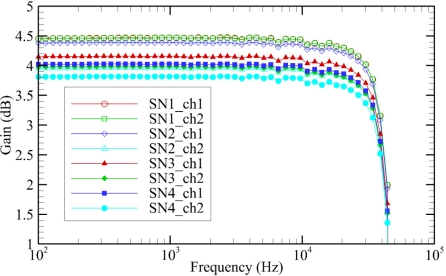
PCM-D50 data recorder frequency response for each channel of each unit tested.

The gain of each PCM-D50 data recorder at each of the frequencies tested, for each channel, are plotted below (Figure 8). The results of the test on the different PCM-D50 data recorders indicate that the gain is slightly more variable than that of the hydrophone signal processing boards. The average gain was 4.15 dB, with a standard deviation of 0.25 dB. The cutoff frequency for the data recorders was very consistent, with a value of 44.3 kHz and a standard deviation of only 6 Hz. This cutoff frequency is very close to the Nyquist frequency of 48 kHz, which is the largest possible frequency that can be measured with a 96-kHz sampling rate.

### 4.3. Complete Underwater Sound Recorder System

**Figure 9.**
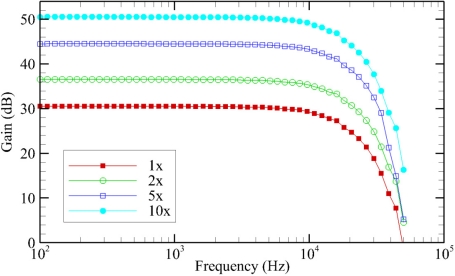
Underwater Sound Recorder system average frequency response for each gain setting.

The average gain of the USR system at each of the frequencies tested, for each of the gain settings on the hydrophone signal processing board, are plotted below (Figure 9). As expected, the gain of the complete USR system was very similar to the gain calculated from the sum of the gain values for the hydrophone signal processing board and the PCM-D50 data recorder. The average difference between the actual system gain and the calculated gain was only 0.02%. Such a small difference helps to strengthen the validity of the tests. The cutoff frequencies of the complete USR systems were on average about 0.5 kHz less than those of the hydrophone signal processing boards alone, with an average value of 14.6 kHz.

### 4.4. Tank Testing

**Figure 10.**
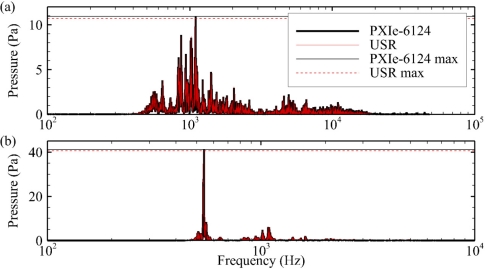
Sample spectrum comparisons from the tank testing. (**a**) Underwater explosion; (**b**) whale call.

**Figure 11.**
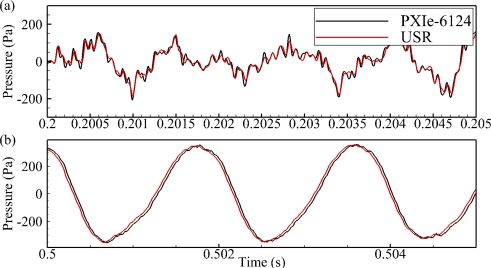
Sample zoomed-in comparisons from the tank testing. (**a**) Underwater explosion; (**b**) whale call.

Overall, the measurements of the two waveforms using the USR systems and the PXIe-6124 data acquisition card compared very well. The measurements of the whale call (Table 1) agreed more closely than the measurements of the underwater explosion (Table 2). This could be a result of the fact that the bandwidth of the whale call is much lower than that of the underwater explosion. A sample spectrum from the measurements of both the whale call and the underwater explosion (Figure 10) as well as a sample of the zoomed-in whale call and underwater explosion waveforms measured (Figure 11) are shown below. The overall average percentage difference of the whale call measurements was 1.4%; the 2× gain setting had the largest average difference of 4.7%. For the underwater explosion measurements, the overall average percentage difference was 3.0%, with the 2× gain setting having the largest average difference of 6.1%.

### 4.5. Case Study

**Figure 12.**
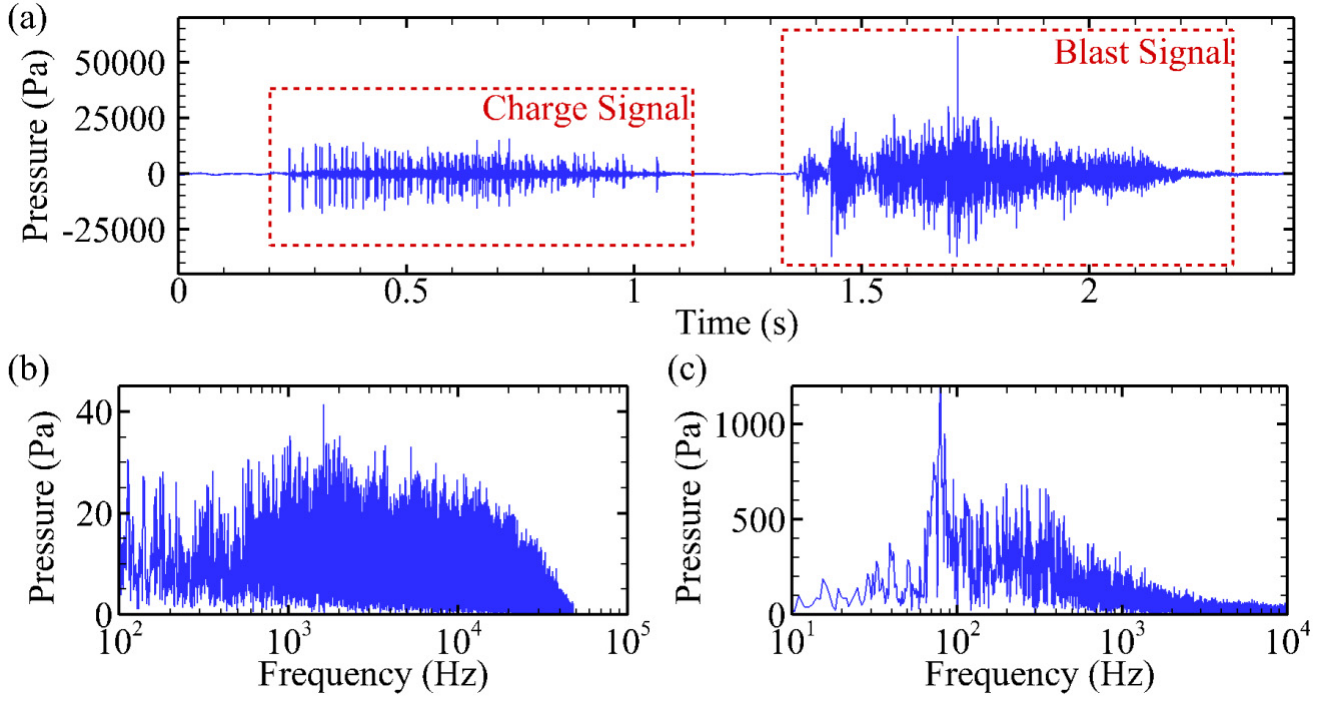
Sample underwater rock blasting measurement. (**a**) Charge and blast signal waveforms; (**b**) charge signal spectrum; (**c**) blast signal spectrum.

For each blasting event, the waveforms recorded consisted of both the explosive charge signal and the main blast signal. From each measurement, the following parameters were calculated from the recorded charge and blast signals: peak positive pressure (kPa), peak negative pressure (kPa), peak absolute pressure (kPa), root mean square (RMS) pressure (kPa), main frequency (Hz), main frequency amplitude (kPa), and duration (s). In addition, the total RMS pressure (kPa) and total power were estimated from the combination of the detonator and explosive charge signals. The charge and blast signal waveforms, as well as the charge and blast signal spectra normalized to physical units of pressure are plotted below (Figure 12).

The ability to record sound and pressure waves with exposure cages was critical for monitoring underwater blasting activities during the winter months in the Pacific Northwest where fog, swift murky water, and heavy vessel traffic complicated the efforts to conduct visual animal surveys. Of the 24 potential sound and pressure wave attributes processed, blast maximum positive pressure (ranging from 14.5 to 163 kPa) was the most significant when regressed against frequency of injury per individual fish (0 to 8 injuries). At lower blast maximum positive pressures, such as 14.5 to 32.78 kPa, 0 to 2 injuries per fish were common. At higher blast maximum positive pressures, such as 103 to 163 kPa, 3 to 8 injuries per fish were observed. The severity or physiological cost associated with each injury type significantly increased with blast maximum positive pressure as well. For example, mild injuries, such as enlarged capillary beds and hematomas, comprised more of the total injuries per fish recorded at lower blast pressures than at higher blast pressures. Conversely, severe injuries, such as hemorrhaging livers and swim bladders, comprised more of the total injuries per fish recorded at higher blast pressures than at lower blast pressures. The resultant relational data between sound pressure level (dose) and fish injuries (response) will improve scientists’ and regulators’ understanding of the effects of underwater explosion on tissue damage, lethality, and estimation of juvenile fish take (see Carlson *et al*. 2011 for full report).

## 5. Conclusions

The primary objective of this study was to design, build, and test a portable, watertight, and user-friendly device to record underwater acoustic signals in potentially extreme environments. This was accomplished by designing the Underwater Sound Recorder (USR). The device allows up to 1 h and 55 min of data to be collected from two sensors simultaneously. The USR filters out frequency components above 15 kHz and lets the total system gain be adjusted to 30 dB, 36 dB, 44 dB, or 50 dB independently for each sensor.

The compact USR enables the collection of underwater acoustic field measurements. To increase system versatility, two versions of the USR were designed and built. Both versions feature identical components housed in a watertight enclosure. The primary difference is that one version is designed to be completely submersed in water and the other is intended for surface-side operation. The submersible version allows the device to be connected to two hydrophones and deployed to a maximum depth of 300 m. This makes the submersible version appropriate for applications where it is desirable to collect long-duration measurements at depths that would require very long hydrophone cables or extension cables, which can introduce additional noise to the measurements. The non-submersible version is better suited for applications in which the underwater event to be monitored has a relatively short duration and a known start and end time; with the non-submersible version, researchers can use the remote control module and USB connector to easily start recording, stop recording, and extract the data. Because the non-submersible version is still watertight, it can be safely operated in a boat or from the shore in nearly all weather conditions.

Tests performed in the laboratory using a data acquisition system to send single-frequency sinusoidal voltages directly to each component have verified that the device operates as designed and performed as well as larger commercially available data acquisition systems, which are not suited for field use. On average, the designed gain values differed from the actual measured gain values by only 0.35 dB. For the four USR systems tested, the average standard deviation of the gain was 0.27 dB. The hydrophone signal processing board accounted for only 7% of the variability between the different systems, with the off-the-shelf Sony PCM-D50 data recorder accounting for the remaining 93%. The results from the tank testing verified that the USR was able to obtain peak pressures in the frequency domain that were very similar to the peak pressures obtained by the National Instruments data acquisition system used.

The case study performed using a prototype of the USR involved recording the sound and pressure waves of several underwater rock blasting events while simultaneously exposing juvenile Chinook salmon and rainbow trout held in cages to the same sound and pressure waves. Necropsies were performed on the exposed fish to identify 77 potential external and internal injuries. Of the 24 potential sound and pressure wave attributes processed, blast maximum positive pressure was the most significant when regressed against frequency of injury per individual fish. The USR was crucial to obtaining the results, which help further the understanding of the effects of underwater explosions on the tissue damage, lethality, and estimation of juvenile fish take.

## Figures and Tables

**Table 1.** Percentage difference between the results from the PXIe-6124 measurements and the Underwater Sound Recorder measurements for the whale call waveform.

	**1× Gain**	**2× Gain**	**5× Gain**	**10× Gain**
**Average**	0.3%	4.7%	0.3%	0.3%
**Std. Dev.**	0.5%	0.6%	0.3%	0.5%

**Table 2.** Percentage difference between the results from the PXIe-6124 measurements and the Underwater Sound Recorder measurements for the underwater explosion waveform.

	**1× Gain**	**2× Gain**	**5× Gain**	**10× Gain**
**Average**	2.5%	6.1%	1.9%	1.5%
**Std. Dev.**	1.7%	1.2%	0.8%	1.0%
	
